# Targeted enrichment of ^28^Si thin films for quantum computing

**Published:** 2020

**Authors:** K Tang, H S Kim, A N Ramanayaka, D S Simons, J M Pomeroy

**Affiliations:** 1 Department of Materials Science and Engineering, University of Maryland, College Park, Maryland 20740, United States of America; 2 National Institute of Standards and Technology, Gaithersburg, Maryland 20899-8423, United States of America; 3 Department of Electrical and Computer Engineering, University of Maryland, College Park, Maryland 20740, United States of America

**Keywords:** isotope enrichment, semiconductor materials, quantum information, molecular beam epitaxy, thin films

## Abstract

We report on the growth of isotopically enriched ^28^Si epitaxial films with precisely controlled enrichment levels, ranging from natural abundance ratio of 92.2% all the way to 99.99987% (0.83 × 10^−6^ mol mol^−1 29^Si). Isotopically enriched ^28^Si is regarded as an ideal host material for semiconducting quantum computing due to the lack of ^29^Si nuclear spins. However, the detailed mechanisms for quantum decoherence and the exact level of enrichment needed for quantum computing remain unknown. Here we use hyperthermal energy ion beam deposition with silane gas to deposit epitaxial ^28^Si. We switch the mass selective magnetic field periodically to control the ^29^Si concentration. We develop a model to predict the residual ^29^Si isotope fraction based on deposition parameters and measure the deposited film using secondary ion mass spectrometry (SIMS). The measured ^29^Si concentrations show excellent agreement with the prediction, deviating on average by only 10%.

## 1. Introduction

Isotopically enriched silicon is regarded as a promising material for semiconductor quantum information due to very long coherence times [1, 2] and its compatibility with the readily available industrial platform. By removing the 4.7% ^29^Si spin-half nuclear spin in natural abundance silicon, qubits can be well isolated from noise sources, e.g., the spectral diffusion of electron spins due to the interaction with the nuclear spin bath. Consequently, great enhancements in coherence times (T_2_) have been observed by numerous research groups, using both silicon-based quantum dots [3] and donor-bound spins. Electron spin coherence times (T_2e_) exceeding seconds [2] and nuclear spin coherence times (T_2n_) approaching an hour [4] have been demonstrated using ^31^P in isotopically enriched ^28^Si. Other donors, such as arsenic [5], bismuth [6, 7] and antimony [8] have also shown great potential in spin qubits.

As interest grows in using isotopically enriched ^28^Si to achieve longer coherence times in quantum information processing, better understanding of the mechanisms behind decoherence in electron spin becomes important. In 1958, Gordon and Bowers first measured T_2_ of electrons bound to lithium and phosphorus donors in isotopically enriched Si with T_2_ = 0.5 ms [9, 10], which was longer than in natural Si. This demonstrated that, in the donor electron spin system, residual ^29^Si contributes significantly to the electron spin decoherence. Recently, theoretical studies using cluster expansion techniques [11–13] by Witzel *et al*., predicted that every order of magnitude increase in isotopic enrichment results in approximately the same order of magnitude increase in the coherence time until limited by non-Si spins. Excellent agreement between the theory and experiment has been shown with bulk ESR measurements, with one measurement done at 0.0005% ^29^Si [2] and others from 0.08% to 99.2% ^29^Si [12]. However, emerging single ^31^P spin measurements in ^28^Si have indicated performance better than predicted [1, 14], motivating additional studies. The discrepancy found between the experiments and theory indicates that the phase space of coherence versus enrichment, especially in the limit of few spins and high isotopic enrichment regimes, remain largely unknown.

As a result, a specific need exists for enriched ^28^Si to have different, targeted values of enrichment to study the dependence of quantum coherence time on residual ^29^Si concentration. Although various research groups have been able to make isotopically enriched ^28^Si [15–19] (explained in detail in [20]), the ability to precisely predict and control the residual ^29^Si isotope fraction within ^28^Si has not yet been demonstrated. The discreteness and the limited number of the enrichment levels available within this community make a detailed determination of the optimal enrichment difficult to accomplish.

In this article, we present a method that allows us to produce ^28^Si with precisely controlled isotopic entrichments. We develop a model that allows us to choose and predict the level of enrichment for our ^28^Si. We deposit ^28^Si thin films with ^29^Si concentrations ranging from our baseline (<1 × 10^−6^ mol mol^−1^) to natural abundance (4.7%) and measure the isotope fractions of the residual ^29^Si and ^30^Si using secondary ion mass spectroscopy (SIMS). The measured enrichments are then compared to the model prediction and show excellent agreement, deviating on average by only 10%.

**Figure 1.**
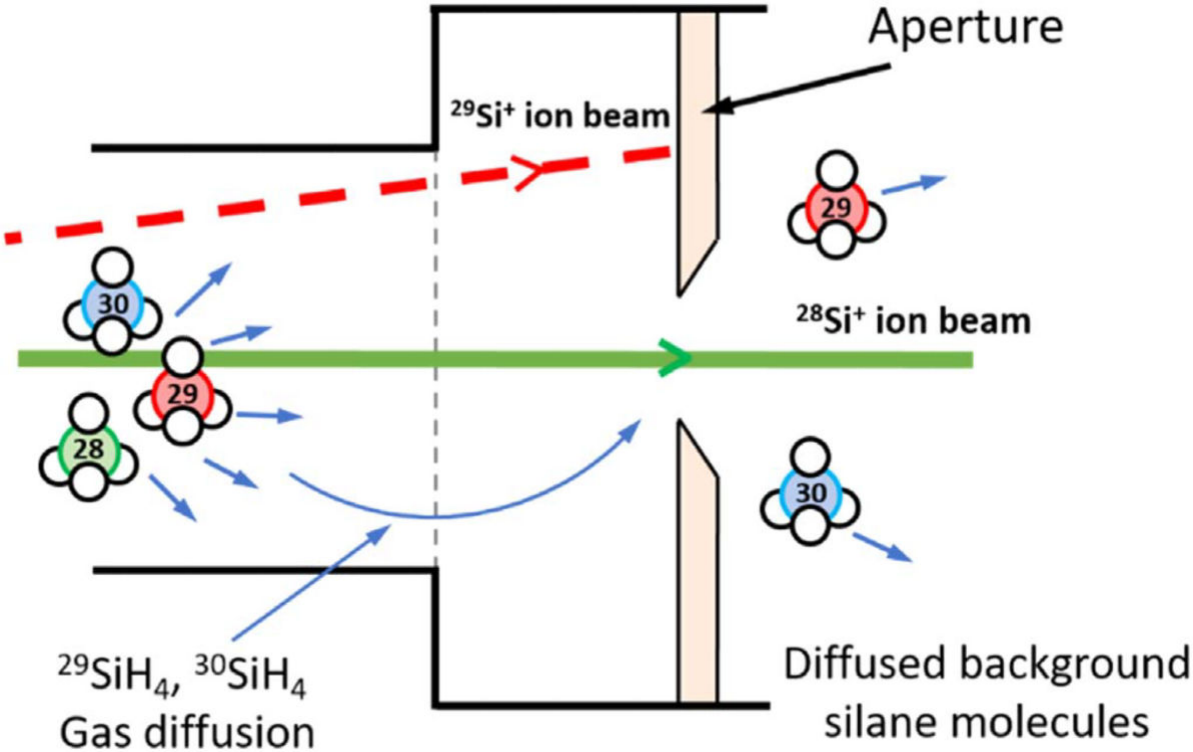
A schematic illustrating the origin of ^28^Si and ^29^Si. Solid green and dashed red lines represent ^28^Si^+^ and ^29^Si^+^ ion beam respectively. During ^28^Si deposition, mass selective magnetic field is tuned such that only ^28^Si^+^ ions can pass through and ^29^Si^+^ ions are blocked by the aperture. Apart from the Si ions, SiH_4_ gas molecules can also pass through the aperture and adhere to the substrate The background silane gas contribution to the film is approximately 10^−6^.

We use a hyperthermal energy ion beam system to deposit isotopically enriched ^28^Si thin films. The experimental setup and capabilities of this deposition system are described in details elsewhere [19,21,22]. In short, ultra-high purity silane gas with natural isotopic abundance is used as source gas and ionized by a UHV Penning-type ion source. The ions are then extracted by an extraction cusp at the end of the ion source and transmitted by electostatic optics into a 90° sector mass analyzer. By tuning the magnetic field of this mass analyzer, only the ions that have certain mass-to-charge ratio (e.g., ^28^Si^+^ that has 28 u/e) are allowed to pass through, as shown in figure 1. Other ions are rejected by the mass analyzer. Beyond this point, ions are refocused and deposited onto a float zone (FZ) natural abundance Si substrate in a UHV deposition chamber.

## 2. Experimental methods

**Figure 2.**
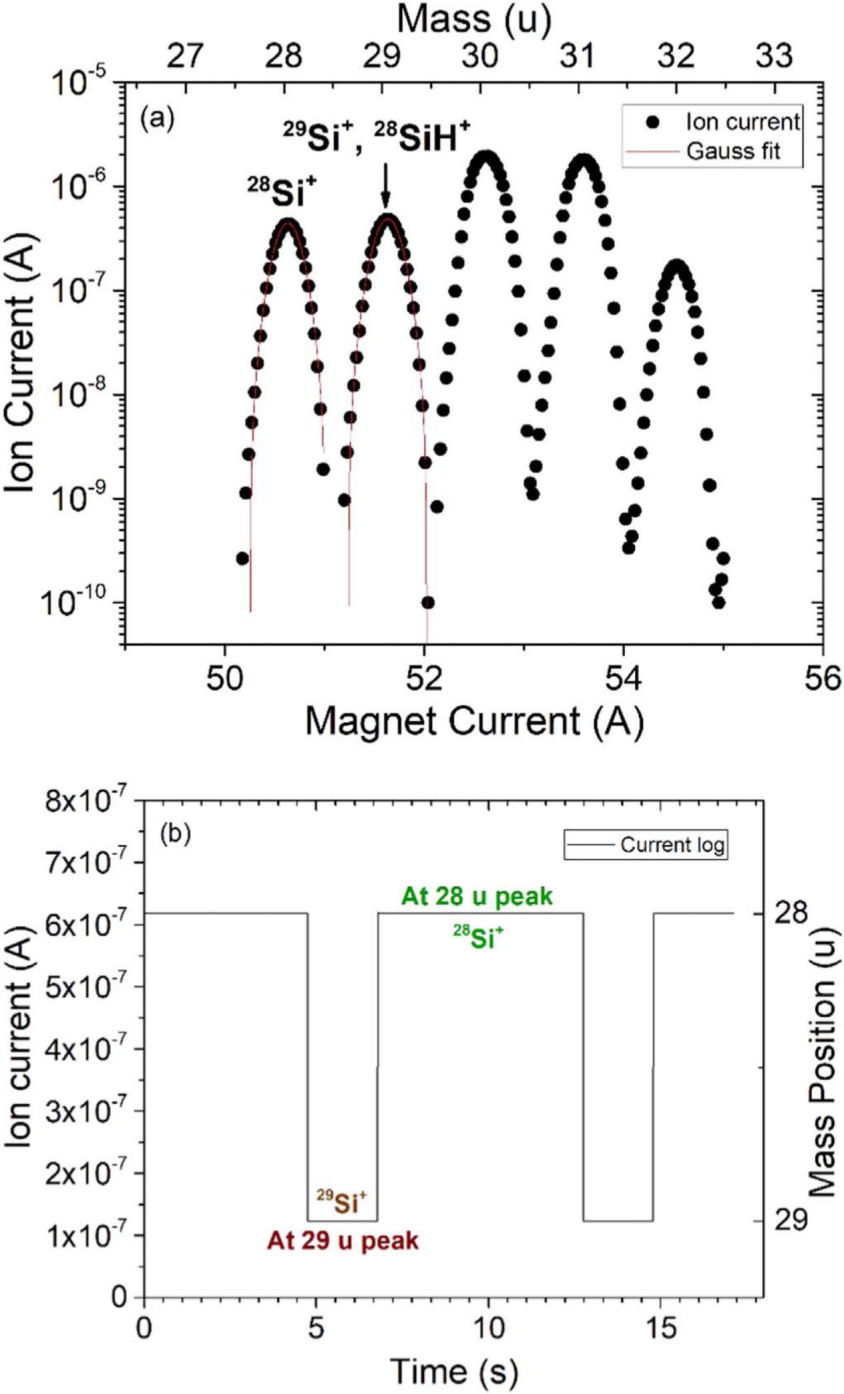
(a) An ion beam mass spectrum used for checking mass resolution and calculating the deposition parameters described in equations (1) and (2). Gaussian fits for both mass 28 u and mass 29 u are shown in red, with a mass separation of 7.4 σ. (b) An example of a current log for targeted enrichment, plotted as the ion current collected at the sample stage versus time. The corresponding mass positions at 28 u and 29 u peaks are also shown on the right. The duty cycle is selected such that the dwelling time at mass 28 u is 75% and the dwelling time at mass 29 u is 25%.

To achieve a targeted enrichment, sources of ^29^Si that can enter the film are studied. As shown in figure 1, even with the magnetic field tuned at 28 u/e, ^29^Si^+^ ions might still pass through the mass selective aperture if the mass resolution is poor. Here we use the mass spectrum to characterize the Si ions. It is generated by monitoring the ion current at the deposition location while tuning the mass analyzer magnetic field. A mass spectrum is shown in figure 2(a) for SiH_4_, where peaks for ^28^Si^+^ ions (mass 28 u), ^29^Si^+^ ions (mass 29 u) and the corresponding ionized hydrides (mass 29 u to 32 u) due to incomplete cracking, can be seen. The mass separation is obtained by fitting the mass peaks with Gaussians. The center of the mass 28 u peak is about 7.4 σ (standard deviation) away from the center of 29 u peak, indicating a lower bound of ^29^Si isotope fraction of 10^−13^ at the 28 u mass position. Another source of ^29^Si comes from the incomplete cracking of SiH_4_ molecules as they can diffuse through the aperture hole and adhere to the sample substrate. In addition, mass 29 u ions might lose energy and fall into the 28 u trajectory during mass filtering. However, since there is no observed scattering tail effect, we assume that all the current from mass 28 u is from ^28^Si^+^. Therefore, the only two active contributors of ^29^Si considered in this paper are the ion beam itself and the diffused background silane gas from the ion source to the deposition chamber.

The experimental concept for targeted enrichment is described in detail here. In previous work, we produced isotopically pure ^28^Si that has a ^29^Si isotope fraction <1 × 10^−6^ mol mol^−1^ by tuning the mass selective magnetic field to be centered on the mass 28 u peak only. However, if we change the magnetic field to the mass 29 u peak for a certain amount of time, we can mix ^29^Si into our ^28^Si film. By controlling the dwelling times Δt_28_ (time spent on the mass 28 u peak) an Δt_29_ (time spent on the mass 29 u peak) periodically, we can control the amount of ^29^Si^+^ deposited onto th esample. This periodic switching is achieved by using a function generator to trigger a square function to control the output of the mass analyzer. The output of the mass analyzer, which contains both the magnet current and the switching periods, determines the mass positions and the dwelling times of the ion beam. Figures 2(a) and (b) demonstrate an example of the control parameters. The peak of the square wave corresponds to the mass 28 u peak (^28^Si^+^ only), at a magnet current of 50.6 A, with an ion current of 620 nA and Δt_28_ of 6 s. The valley of the square wave corresponds to the mass 29 u peak (^29^Si^+^ and ^28^SiH^+^), at a magnet current of 51.6 A, with an ion current of 124 nA and Δt_29_ of 2 s. These parameters would correspond to a ^29^Si isotope fraction of 3 × 10^−3^ mol mol^−1^, with roughly 1 monolayer of Si deposited per cycle.

In this way, by tuning the dwelling times Δt_28_ and Δt_29_, we are able to produce any desired enrichment level, ranging from natural abundance (4.7% ^29^Si) to our baseline (<1 × 10^−6^ mol mol^−1 29^Si). The dwelling time Δt_28_ at mass 28 u, and Δt_29_ at 29 u can be any combination as long as it is within the response time of the analyzer power supply, which is about 2 ms in the range of our interests. However, to ensure the epitaxial quality and homogeneity of the deposited ^28^Si material, Δt_28_ + Δt_29_ should be a short cycle, generally corresponding to a monolayer of material growth. During deposition, we tune the ion beam to its optimum fluence condition, with a SiH_4_ flow rate of 0.02 sccm (corresponding to a chamber pressure of 1.87 × 10^−4^ Pa or 1.4 × 10^−6^ Torr) and a growth rate of about 1.0 to 1.5 mm min^−1^ [22]. Higher growth rate is also achievable using high pressure plasma mode of the ion source, but generally results in a higher surface roughness of the deposited film. The substrate temperature is chosen to be 450 °C, which produces the lowest baseline ^29^Si isotope concentration and highest epitaxial film quality [23] for this experimental setup.

A model is developed to calculate the isotope fractions of the deposited ^28^Si layer, including the contributions from the background silane gas:

(1) $$f^{29}=\frac{\Delta t_{29}\times D_{29}\times A+{}^{29}C_{z}\times (\Delta t_{29}\times D_{29}+\Delta t_{28}\times D_{28})}{\left(\Delta t_{29}\times D_{29}+\Delta t_{28}\times D_{28})\times (1+{}^{28}C_{z}+{}^{29}C_{z}+{}^{30}C_{z}\right)}$$

(2) $$L=(\Delta t_{29}\times D_{29}+\Delta t_{28}\times D_{28})\times (1+{}^{28}C_{z}+{}^{29}C_{z}+{}^{30}C_{z}),$$

where f^29^ is the isotope fraction of ^29^Si, L is the number of monolayer per cycle, D_28_ is the deposition rate of ^28^Si at mass 28 u peak current, D_29_ is the depostion rate at 29 u peak current, A is the atomic percentage of ^29^Si at 29 u peak, which consists both ^29^Si^+^ and ^28^SiH^+^ ions. ^28,29,30^C_z_ are the flux ratios from the background silane diffusion, which can be calculated using the equation derived from [23]:

(3) $${}^{x}C_{z}=\frac{F_{g}\times a_{x}\times s}{F_{g}\times s+F_{i}},$$

where F_g_ is the silane gas flux and F_i_ is the ^28^Si ion flux, s is the effective incorporation fraction and a_x_ is the natural abundance ratio of the corresponding silicon isotopes in SiH_4_. In this experimental setup, since we are using low SiH_4_ pressure mode for ^28^Si deposition, of the background gas contribution is typically <1 × 10^−6^ mol mol^−1 29^Si, which has negligible impact on most of the enrichment levels but is still included in the calculation.

## 3. Results and discussions

In each deposition, typically two or three layers of ^28^Si with different enrichments are grown on one substrate based on the model described above, each with a layer thickness of about 100 nm. The sequence is to choose a target value first, then estimate the value after deposition and finally compare to the measured value using SIMS. It is worth noticing that the ion beam growth condition might change a little during deposition. Therefore, the estimated value calculated after deposition may deviate from the targeted values before deposition, but generally the deviation is small (5.7% on average).

**Figure 3.**
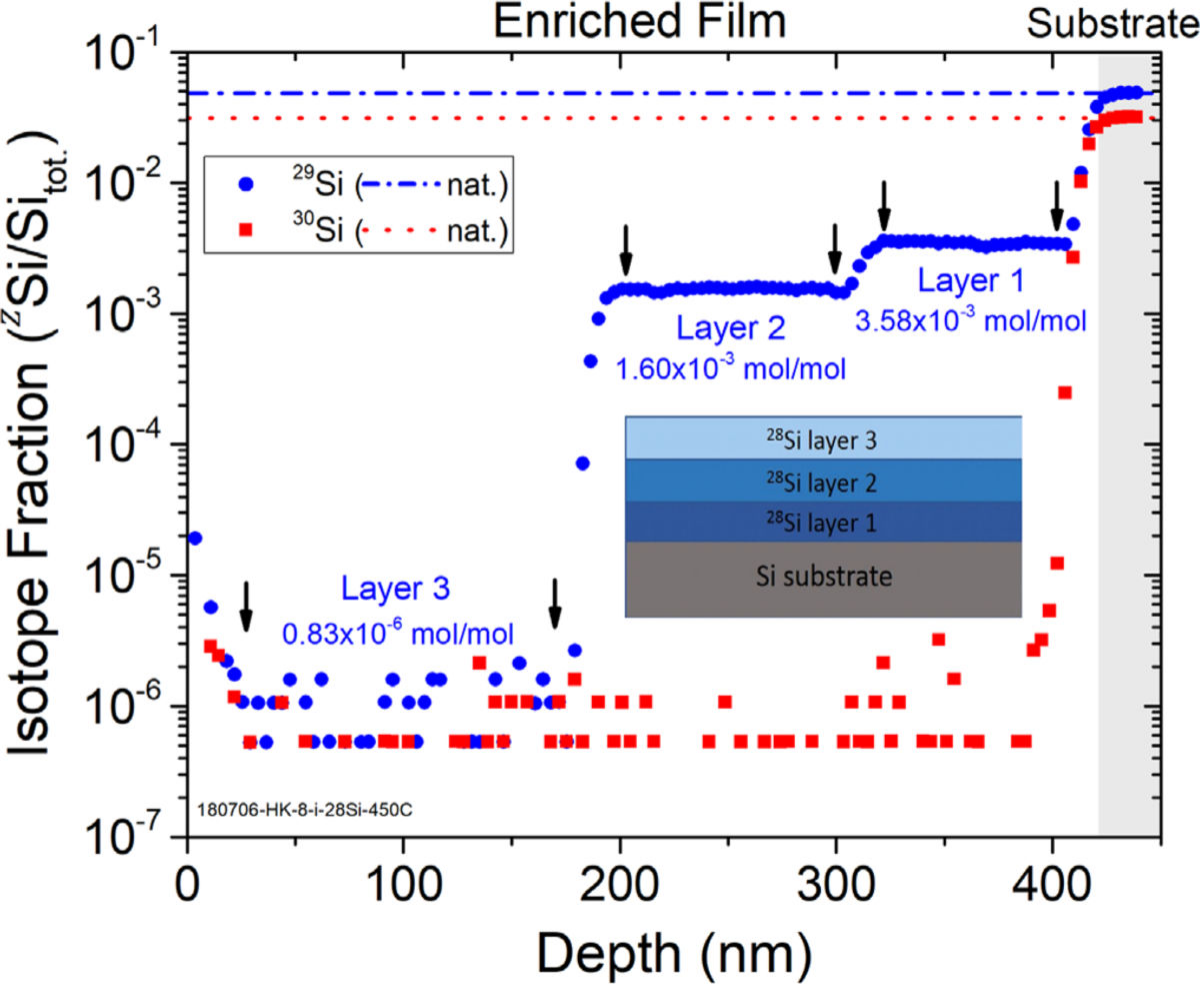
A SIMS depth profile of a targeted enrichment sample. The inset shows a schematic diagram of the targeted enrichment sample layer structures. Usually a few layers with different ^29^Si isotope fractions are deposited on a float-zone silicon substrate and then capped with pure ^28^Si layer. The ^29^Si and ^30^Si isotope fractions are shown in blue dots and red squares, respectively. Natural abundance ratios of ^29^Si and ^30^Si are shown in dashed lines. Three layers can be seen here, corresponding to the three different ^29^Si isotope fractions: (3.58 ± 0.02) × 10^−3^ mol mol^−1^, (1.60 ± 0.01) × 10^−3^ mol mol^−1^ and (0.83 ± 0.09) × 10^−6^ mol mol^−1^.

The isotope fractions of ^28^Si, ^29^Si and ^30^Si as a function of layer thickness in the film and the substrate are measured using SIMS. The isotope measurements were made by a large geometry secondary ion mass spectrometer with a resolving power of 6000 (M/ΔM at 10% of peak maximum). This resolving power is necessary to separate the ^29^Si peak from the ^28^SiH peak that is produced during the SIMS process. Under these conditions, we estimate that less than 10^−5^ of the ^28^SiH signal contributes to the ^29^Si, making it negligible for all samples measured here. Figure 3 shows the SIMS depth profile of one of the targeted enrichment samples, where three different enrichment levels can be distinguished. The SIMS measurements were taken near the center of the ^28^Si deposit, which is usually thickest, to match the parameters used in the model. The average isotope fraction of ^29^Si in surface layer (baseline) is measured to be (0.83 ± 0.09) × 10^−6^ mol mol^−1^, from the range of 30 nm to 170 nm depth. Higher values of ^29^Si and ^30^Si are found from 0 nm to 30 nm, since the sample has been exposed air and to adventitious sources of silicon that release a small amount of boron and silicon in vapor phase, which can land on the sample surface. Furthermore, the 7 keV O_2_^+^ primary ion beam in the SIMS instrument produces a ‘knock-on’ effect that drives the surface atoms forward as the beam erodes below the initial surface, producing a tail. Two subsequent layers are also shown from 170 nm to 310 nm and 310 nm to 417 nm, with an average ^29^Si isotope fraction of (1599 ± 7) × 10^−6^ mol mol^−1^ and (3583 ± 20) × 10^−6^ mol mol^−1^, respectively. The targeted values are 1600 × 10^−6^ mol mol^−1^, with a deviation (compared to the measured value) of 0.06% and 3500 × 10^−6^ mol mol^−1^, with a deviation of 2.4%. As a comparison, the estimated values from the model after deposition are calculated to be (1630 ± 15) × 10^−6^ mol mol^−1^, with a deviation of 1.9% and (3530 ± 30) × 10^−6^ mol mol^−1^, with a deviation of 1.5%, which are quite close to the targeted values.

**Figure 4.**
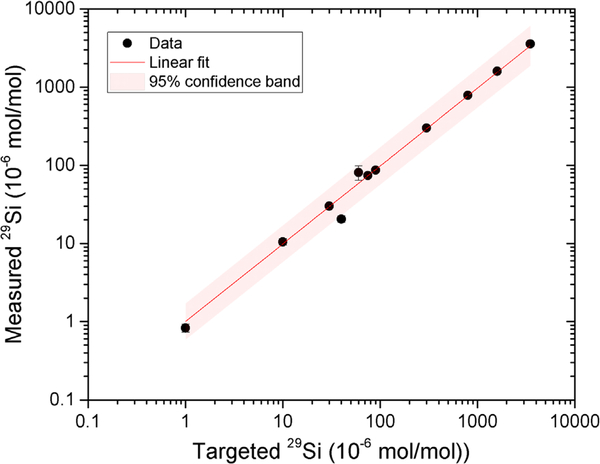
A correlation plot showing the measured ^29^Si isotope fractions as a function of targeted ^29^Si isotope fractions. A linear fit and 95% confidence band are included to assist comparison. An average deviation of 10% has been obtained over a wide range, from 0.83 × 10^−6^ mol mol^−1^ to 3.58 × 10^−3^ mol mol^−1^ of ^29^Si.

The comparison between the targeted and measured ^29^Si isotope fractions is shown in detail in table 1 and a correlation plot of the targeted versus the measured ^29^Si isotope fraction is shown in figure 4, with error bars. In total, 11 targeted enrichment levels have been plotted on a log scale, ranging from 0.83 × 10^−6^ mol mol^−1^ to 3583 × 10^−6^ mol mol^−1^ of ^29^Si. Both a linear fit and a confidence band are included to show the accuracy of the prediction. As shown in the figure, all data points are within 95% confidence band. The average deviation between the targeted and measured enrichments across the entire range of measurements is found to be 10%. The one data point measured at 20 × 10^−6^ mol mol^−1^ the largest deviation from the targeted value and the largest relative uncertainty. This deviation was caused by the ion source, where the ion beam condition was unstable during this deposition compared to others. Better accuracy of the enrichment can be achieved by increasing the stability of the ion source, for example, using a more sputter-resistant material (for example Ti) for the cathodes. Cleaning of the ion source using argon plasma may also be helpful for the stability, since silicon flakes slowly aggregate on the interior of the ion source and cause fluctuation in the plasma region. Another source of uncertainty may come from the location of the ^28^Si spot. Since our ^28^Si deposit is in the shape of a hill instead of flat surface, the measured location might be different from where it has been estimated. Since this work, an ion beam sweeper to smooth out the deposited film has been added. Furthermore, the SIMS measurement uncertainty also acts as a factor, mainly limited by counting statistics, especially at lower ^29^Si concentrations, where the number of counts is dramatically lower compared to higher ^29^Si concentrations. Finally, these films are suspected to suffer from higher levels of chemical contamination than commercial, electronics grade silicon, but ongoing efforts are expected to suppress this contamination and the detailed impact on quantum device performance is unknown.

## 4. Conclusions

In summary, we have reported on a method that allows us to achieve targeted enrichment of the ^28^Si epitaxial thin films. We develop a model to precisely predict and control the residual isotope fraction of the ^29^Si in the film and compare its results to the values measured using SIMS. We find excellent agreement between the targeted and the measured values over a wide range of enrichments, with small deviation of 10% on average. This deviation can be further improved by increasing the stability of our ion source and by using an ion beam sweeper. In comparison to other isotopic enrichment methods, such as chemical vapor deposition (CVD), this ion beam deposition has the advantage of having much lower thermal budget, making it suitable for qubit architectures that requires low temperature processing, e.g., STM fabricated single atom qubits. We believe this is an important step forward to explore the qualifying metric for ‘quantum grade’ silicon in terms of enrichments.

## Figures and Tables

**Table 1.** A comparison between the target, estimated and measured ^29^Si isotope fractions. The deviation shown here are between the target and the measured values. The total deviation on average is (10.4 ± 5.0) %.

Target (10^−6^ mol mol^−1^)	Estimated from deposition (10^−6^ mol mol^−1^)	Measured by SIMS (10^−6^ mol mol^−1^)	Deviation
1	0.7	0.83	17%
10	9.9	10.5	5%
30	34.1	30	0%
40	40.7	20.5	48.75%
60	62.1	81	35%
75	77	74	1.33%
90	88.1	87	3.33%
300	316	300	0%
800	797	784	2%
1600	1630	1599	0.06%
3500	3530	3583	2.37%
